# Factors Associated with Unprotected Anal Intercourse among Men Who Have Sex with Men in Liaoning Province, China

**DOI:** 10.1371/journal.pone.0050493

**Published:** 2012-11-27

**Authors:** Jie Liu, Bo Qu, Moses C. Ezeakile, Yang Zhang

**Affiliations:** Faculty of Health Statistics, School of Public Health, China Medical University, Shenyang, China; Yale School of Public Health, United States of America

## Abstract

**Background:**

HIV prevalence among men who have sex with men (MSM) has increased rapidly. MSM may play a bridging role in the spread of HIV and other STDs from the high-risk population to the general population. Interventions to reduce high-risk behavior are the key to controlling the spreading of HIV in the MSM population and the primary strategy for reducing the spread of AIDS in China. The purpose of the study was to examine the demographic characteristics of MSM, evaluate the HIV-related knowledge of MSM, and identify factors associated with unprotected anal intercourse (UAI) among MSM to make recommendations for future research.

**Methodology/Principal Finding:**

A cross-sectional survey was conducted among 293 MSM in Fushun and Huludao City, China. A total of 91 participants (34.0%) reported engagement in UAI with a male partner during the previous six months. The results of univariate analysis showed that UAI was associated with older age, lower levels of education, less knowledge about HIV, and not receiving condoms, lubricant, peer education, AIDS counseling, STD checks, and informational materials (*p*<0.05). In a multivariate logistic regression model, awareness of the major HIV transmission routes (OR = 2.191; 95% CI: 0.869 to 5.524), receiving condoms (OR = 2.164; 95% CI: 1.149 to 4.076), receiving peer education (OR = 2.632; 95% CI: 1.566 to 4.426), and AIDS counseling (OR = 2.347; 95% CI: 1.260 to 4.372) were independently associated with a lower risk of UAI.

**Conclusions/Significance:**

The study suggested that UAI could be decreased by improving education about AIDS, increasing the promotion of voluntary counseling and testing (VCT), and improving the accessibility and convenience of service.

## Introduction

HIV/AIDS in China has entered a critical stage of rapid and widespread increase, and of particular concern is the increasing HIV prevalence among men who have sex with men (MSM) [1], [2]. The percentages of newly reported HIV cases attributable to MSM in China were 0.2% in 2001, 7.3% in 2005, 12.2% in 2007, and 32.5% in 2009 [3]. Sexual transmission, including both heterosexual and homosexual transmission, has become the dominant route of HIV infections, accounting for more than 70% of the estimated new infections in 2009 [4]. Traditional Chinese culture does not openly endorse MSM behaviors. MSM behaviors are highly unacceptable, and there is strong social pressure against the behavior of MSM [5], [6]. MSM may play a bridging role in the spread of HIV and other STDs from the high-risk population to the general population. A study showed approximately one-half of MSM reported also having had sex with a woman and one-third having been married to a woman [7].

UAI among MSM have been documented in different parts of the world, including China [8], [9]. UAI place the Chinese MSM at higher risk for HIV infection [10]. Evidence from studies showed that condom use among MSM remains at a very low level [8]–[16]. It has been broadly reported in China that most MSM had sex with casual sexual partners and never used a condom [7], [17]. To control the situation, improvement on public health programs in recent years were made by strengthening the education programs that promote the use of condoms [18]–[20]. However, due to cultural stigma and social discrimination associated with homosexual behavior in China, MSM have to hide their sexual orientation and have little access to the public health care system or to educational information [21]. Therefore, health interventions to reduce UAI are the key to controlling the spreading of HIV in MSM and the primary strategy to slow the spread of AIDS in China. The purpose of the study was to examine the demographic characteristics of MSM, evaluate the HIV-related knowledge of MSM, and identify factors associated with UAI among MSM in Liaoning Province, China, in order to make recommendations for future research.

**Table 1:** Main characteristics of MSM in Liaoning province, China (*n* = 268).

Variables	UAI		*p*	Total
	Yes	No		
**Marital status**			0.427*	
Unmarried	56(31.5%)	122(68.5%)		178(66.4%)
Married	27(40.3%)	40(59.7%)		67(25.0%)
Divorced or widowed	8(34.8%)	15(65.2%)		23(8.6%)
**Ethnicity**			0.124*	
Han	84(35.6%)	152(64.4%)		236(88.1%)
Other	7(21.9%)	25(78.1%)		32(11.9%)
**Education** *			0.004* ^1^	
Primary and below	5(83.3%)	1(16.7%)		6(2.2%)
Middle school	28(45.9%)	33(54.1%)		61(22.8%)
Secondary school or high				
school	24(25.5%)	70(74.5%)		94(35.1%)
College	34(31.8%)	73(68.2%)		107(39.9%)
**Vocation**			0.352*	
Unemployed	19(33.3%)	38(66.7%)		57(21.3%)
Students	9(22.5%)	31(77.5%)		40(14.9%)
White collar	18(34.0%)	35(66.0%)		53(19.8%)
Blue collar	45(38.1%)	73(61.9%)		118(44.0%)
**Monthly income**			0.697*	
No income	24(29.3%)	58(70.7%)		82(30.6%)
∼1000 Yuan	22(41.5%)	31(58.5%)		53(19.8%)
1000–2000 Yuan	31(34.1%)	60(65.9%)		91(34.0%)
2000–3000 Yuan	9(32.1%)	19(67.9%)		28(10.4%)
3000∼	5(35.7%)	9(64.3%)		14(5.2%)
**Sexual orientation**			0.276*	
Gay	44(31.0%)	98(69.0%)		142(53.0%)
Bisexual	47(37.3%)	79(62.7%)		126(47.0%)
**Sexual orientation disclosed to at least one relative or friend**			0.490*	
Yes	6(27.3%)	16(72.7%)		22(8.2%)
No	85(34.6%)	161(65.4%)		246(91.8%)
**Mode of recruitment**			0.590*	
Bar/nightclub based	6(30.0%)	14(70.0%)		20(7.5%)
Bath/Saunas based	21(36.2%)	42(66.8%)		63(23.5%)
Park/latrine based	24(41.4%)	34(58.6%)		58(21.6%)
Internet based	40(31.5%)	87(68.5%)		127(47.4%)

UAI = unprotected anal intercourse.

* *χ* ^2^ test.

* ^1^ Fisher’s exact test.

**Table 2:** The HIV/AIDS related transmission knowledge of MSM populations.

HIV/AIDS transmission routes question	UAI		*p*	Total
	Yes	No		
**Could blood or blood product transfusions tainted with HIV cause infection with HIV?**			0.007* ^1^	
Yes	83(32.3%)	174(67.7%)		257(95.9%)
No	6(85.7%)	1(14.3%)		7(2.6%)
Don’t know	2(50.0%)	2(50.0%)		4(1.5%)
**Could sharing needles for drug use with someone who has HIV or AIDS cause HIV infection?**			0.137* ^1^	
Yes	83(32.8%)	170(67.2%)		253(94.4%)
No	7(50.0%)	7(50.0%)		14(5.2%)
Don’t know	1(100.0%)	0(0.0%)		1(0.4%)
**Can a pregnant woman with HIV give the virus to her baby?**			0.017* ^1^	
Yes	82(32.2%)	173(67.8%)		255(95.1%)
No	7(70.0%)	3(30.0%)		10(3.7%)
Don’t know	2(66.7%)	1(33.3%)		3(1.1%)
**Is it risky to eat in a restaurant where the cook has HIV?**			0.021* ^1^	
Yes	12(57.1%)	9(42.9%)		21(7.8%)
No	76(31.4%)	166(68.6%)		242(90.3%)
Don’t know	3(60.0%)	2(40.0%)		5(1.9%)
**Can HIV be spread by mosquitoes or other insects?**			0.134*	
Yes	28(40.6%)	41(59.4%)		69(25.7%)
No	58(30.5%)	132(69.5%)		190(70.9%)
Don’t know	5(55.6%)	4(44.4%)		9(3.4%)

UAI = unprotected anal intercourse.

* *χ* ^2^ test.

* ^1^ Fisher’s exact test.

**Table 3:** The utiliaztion of Voluntary Counseling and Testing (VCT) service.

Item	UAI		*p*	Total
	Yes	No		
Received condoms			0.000*	
Yes	46(25.3%)	136(74.7%)		182(67.9%)
No	45(52.3%)	41(47.7%)		86(32.1%)
Received lubricants			0.000*	
Yes	41(25.3%)	121(74.7%)		162(60.4%)
No	50(47.2%)	56(52.8%)		106(39.6%)
Received peer education			0.000*	
Yes	36(24.3%)	112(75.7%)		148(55.2%)
No	55(45.8%)	65(54.2%)		120(44.8%)
Received STD (HIV/syphilis) testing			0.025*	
Yes	14(22.2%)	49(77.8%)		63(23.5%)
No	77(37.6%)	128(62.4%)		205(76.5%)
Received HIV testing			0.016*	
Yes	10(19.6%)	41(80.4%)		51(19.0%)
No	81(37.3%)	136(62.7%)		217(81.0%)
Received syphilis testing			0.030*	
Yes	9(20.0%)	36(80.0%)		45(16.8%)
No	82(36.8%)	141(63.2%)		223(83.2%)
Received AIDS advisory			0.000*	
Yes	19(15.6%)	103(84.4%)		122(45.5%)
No	72(49.3%)	74(50.7%)		146(54.5%)
Received AIDS information materials			0.001*	
Yes	45(26.5%)	125(73.5%)		170(63.4%)
No	46(46.9%)	52(53.1%)		98(36.6%)

UAI = unprotected anal intercourse.

* *χ* ^2^ test.

**Table 4:** Multivariate logistic modeling of factors associated with UAI among MSM.

Variables	*OR*	*95% CI*	*P*
appropriate responses to the 5 HIV-related transmission routes items≥4			
Yes	1		
No	2.191	0.869–5.524	0.046
Received condoms			
Yes	1		
No	2.164	1.149–4.076	0.017
Received peer education			
Yes	1		
No	2.632	1.566–4.426	0.041
Received AIDS advisory			
Yes	1		
No	2.347	1.260–4.372	0.007

OR = odds ratio; CI = confidence interval; UAI = unprotected anal intercourse.

## Methods

### Participants and Procedures

A cross-sectional study was conducted in Fushun and Huludao City, Liaoning Province, China, from June to October, 2009. Respondents were recruited from venues such as gay bars, gay saunas, and parks where MSM meet one another. UAI was defined as unprotected anal intercourse with any male partners and was assessed by asking men whether they had engaged in any receptive or insertive anal sex with any male partners in the past six months and, if so, whether condoms had been used.

The respondents underwent a face-to-face interview with a standardized questionnaire, which was initially drafted based on the contents formulated by relevant previous studies [22], [23]. The questionnaire was divided into two parts. The first part included questions dealing with socioeconomic characteristics, and the second part was about knowledge, service utilization, and sexual-related behaviors. HIV/AIDS-related knowledge was measured with a 5-item set of questions covering the routes of viral transmission. The knowledge questionnaire was reviewed for content validity by five experienced Chinese HIV/AIDS epidemiology professors. Respondents were given the option to state whether each statement was true or false or that they were uncertain. Service utilization was measured with six items. Sexual behavior information included two items. HIV and syphilis testing were performed on blood samples drawn from the participants. Blood specimens were tested for the HIV antibody by enzyme-linked immunosorbent assay (ELISA) (Beijing Wantai Biologic Medicine Co., China). Reactive ELISA specimens were confirmed by a HIV-1/2 Western Blot immune assay (HIV Blot 2.2 WB; Genelabs Diagnostics, Singapore). Samples testing positive in both tests were considered HIV-positive. Syphilis antibodies were detected by ELISA (Beijing Jinhao Biologic Production Co., China).

Before formal investigation, the interviewers were trained together to understand the purpose and meaning of the investigation, be familiar with the contents and the courses, and be skilled in the interview. They were also trained to be able to explain any question in the questionnaire on the spot. The interviewers signed a form pledging that they had clearly explained the details of the study to the respondents. The interviews were anonymous; no identifying information was collected. A verbal informed consent was obtained from each respondent before the anonymous face-to-face interview. Participation in the study was completely voluntary, and participants had the option of declining to answer specific questions or of leaving the entire questionnaire blank if they did not wish to participate. The survey is anonymous and voluntary, so no written consent was obtained. The study protocol was approved by the bioethics advisory commission of China Medical University.

During the investigation, to ensure the quality of the questionnaire, the questionnaires had to be filled out face to face. After completion of the questionnaires, specially trained personnel inspected the questionnaires, identified the questionnaires that were filled out with non-standard and ambiguous answers, and found the investigators and respondents for timely verification. An incentive of 50 Yuan (equivalent to about 7 USD) was given to respondents as compensation for the time spent being interviewed.

### Data Analysis

Data was double entered and evaluated for congruency using the EpiData software (version 6.4; EpiData Association; Odense, Denmark). SPSS software (Version 16.0; SPSS Inc, Chicago, IL) was used for analysis. *χ* ^2^ or Fisher’s exact test was used to examine independent associations between variables and UAI in the past six months. Variables with a *p*-value <0.20 at univariate results were considered eligible for the multivariate analysis. A backward procedure based on the Wald test was used to select significant variables in the multivariate model. The significance level was fixed at α = 0.05.

## Results

### Socioeconomic Characteristics

A total of 293 MSM were surveyed for the study. In total, 268 MSM completed the questionnaire (response rate: 91.4%). All MSM were aged between 19 and 72 years (mean ± SD: 32.5±11.1 years). Age at first sexual intercourse ranged from 10 to 45 years (mean ± SD: 20.5±4.1 years). In terms of sexual orientation, 53.0% identified themselves as gay and 47.0% as bisexual. Among all respondents, 25.0% were married. The age-specific prevalence of marital status was as follows: less than 30 years old (2.0%), more than 30 years old (60.3%), and more than 40 years old (68.8%). 27.6% of the respondents reported having also had sex with women in the past 6 months, with 38.7% reporting no condom use and 46% reporting inconsistent condom use. 59.5% of the respondents reported having not used a condom in their last sexual intercourse with a female within the past 6 months.

A total of 91 participants (34.0%) reported UAI with any male partner during the previous six months. Reporting UAI in the last six months was significantly associated with older age (*p*<0.05), but was not significantly influenced by the age at first sexual intercourse (*p*>0.05). Bivariate associations between the respondents’ socioeconomic characteristics and UAI in the past six months were summarized in Table 1.

### HIV/AIDS Knowledge

A total of 95.9%, 94.4% and 95.1% of the subjects were aware that HIV was transmitted by “transfusions tainted with HIV”, “sharing intravenous needles”, and “from mother to child”, respectively. The proportion of subjects that correctly selected the non-transmission routes was lower: e.g. “mosquitoes or other insects” (70.9%)”. Knowledge of the items “blood transmission”, “from mother to child” and non-transmission routes “risky to eat in a restaurant where the cook has HIV” was significant for UAI (*p*<0.05). Details of HIV/AIDS knowledge were shown in Table 2.

### HIV/AIDS Service Utilization

A total of 67.9%, 60.4% and 55.2% of the subjects received condoms, lubricant, and peer education, respectively. The proportions receiving STD checks, AIDS counseling, and informational materials were 23.5%, 55.6%, and 63.4%, respectively. In univariate analyses, the following factors were found to be associated with UAI: receiving condoms, lubricant, and peer education, and receiving STD checks, AIDS counseling, and informational materials (*p*<0.05). The results of social service utilization were illustrated in Table 3.

### Factors Associated with UAI among MSM in the Multivariate Analysis

In the univariate analyses (Table 1 and Table 2), reporting UAI in the last six months was significantly associated with older age, lower levels of education, less knowledge about HIV, and not receiving condoms, lubricant, peer education, AIDS counseling, STD checks, and informational materials (p<0.05). Logistic regression was used to identify factors associated with UAI during the previous six months. Significant factors affecting UAI included in the model were as follows: awareness of the major HIV transmission routes (OR = 2.191; 95% CI: 0.869 to 5.524), receipt of condoms (OR = 2.164; 95% CI: 1.149 to 4.076), receipt of peer education (OR = 2.632; 95% CI: 1.566 to 4.426), and AIDS counseling (OR = 2.347; 95% CI: 1.260 to 4.372). The results of the logistic regression analysis were shown in Table 4.

## Discussion

The high prevalence of UAI between MSM and their female partners creates greater health risks in society due to the increase in the spread of HIV. Our results suggested that 47.0% of MSM respondents considered themselves to be bisexual, and about 60% or greater of the MSM respondents who were over 30 years old were married. During sexual intercourse with a female in the past 6 months, 38.7% of the respondents did not use a condom, and only 15.3% used condoms, which may raise the possibility of the MSM population being a bridge population for the spread of HIV from the high-risk population to their female partners and to the general population. Therefore, effective strategies for prevention are needed for reducing the bridging role of the MSM population.

In our study, 34.0% of MSM reported engaging in UAI in the previous six months. The prevalence of UAI among MSM in Fushun and Huludao City was similar to that in other second tier cities and relatively lower than the prevalence of UAI in Beijing (49.8%), Shanghai (57%), and Guangzhou (54.7%), China [24]–[26]. Migrant MSM and those engaging in commercial sex might be particularly at risk for HIV, as seen by their higher levels of UAI in the developed cities above [22], [27].

The respondents in this study have a low acceptance rate for sexually-transmitted disease (STD) testing (23.5%) and AIDS counseling (45.6%). A potential explanation is that they fear the possibility of having a positive test result and losing family or friends [28], [29] and someone would find out about their test results [30]. The fear of discrimination also represents an important barrier to testing and counseling [31], [32]. We also found that only 8.2% of MSM disclosed their sexual orientation to relatives or friends. Therefore, effective prevention programs, including condom promotion and distribution and HIV/AIDS advisory programs, should be implemented to reduce the prevalence of UAI.

In the multivariate logistic regression model, we found that awareness of the major HIV transmission routes, receiving peer education, receiving condoms, and AIDS counseling were independently associated with a lower risk of UAI (*p*<0.05). Participants who did not know HIV-related transmission routes were two times more likely to engage in UAI. This is similar to other recent reports [33]–[36]. This underlined the need to explore more specifically designed education methods to target the most vulnerable group.

Peer education was significantly related to UAI in the past six months, which is consistent with other recent reports. Studies reported that peer education activities had a significant impact on sexual behavior [25], [37]–[40]. Homosexual activities are not illegal in China, but they are not socially acceptable. Education regarding safer sex practices among MSM is a huge challenge since they are socially marginalized and hard to reach. Peer-implemented education was better accepted, as the messages were more adapted to individuals’ needs. Peer-based interventions centered on MSM practices were more adapted to the needs of MSM.

In this study, we found that those MSM who had not received condoms had a 2.2 times greater risk of engaging in UAI than those who had received condoms. Studies suggested a positive association between condom use in male-to-male anal intercourse and access to HIV preventive actions [41]–[46]. The respondents receiving condoms were more likely to use a condom, which indicated that the risky sexual behavior associated with HIV infection could be constrained, in part, by increasing condom distributions.

The study has a number of limitations. Firstly, responses may be subjected to reporting bias because MSM are a hidden population and certain MSM behaviors may be discriminated against. Secondly, our study was conducted in a limited urban area in Liaoning Province, China. Further work that focuses on extending the research into the broader areas in northern China would be valuable.

### Conclusion

In conclusion, for the MSM population in Fushun and Huludao City, UAI may be decreased by improving education about AIDS, increasing the promotion of voluntary counseling and testing (VCT), and improving convenient accessibility to relevant health services.
